# Impact of Production Systems on the Levels of Vitamin E, β-Carotene, and Cholesterol in the Liver of Cattle Raised in the Eastern Amazon

**DOI:** 10.3390/foods13111595

**Published:** 2024-05-21

**Authors:** Adriny dos Santos Miranda Lobato, Jamile Andréa Rodrigues da Silva, Thomaz Cyro Guimarães de Carvalho Rodrigues, André Guimarães Maciel e Silva, Andrea Viana da Cruz, Ana Paula Damasceno Ferreira, Mónica Mendes Costa, António Marcos Quadros Cunha, Vanessa Vieira Lourenço-Costa, Antônio Vinícius Corrêa Barbosa, José António Mestre Prates, Welligton Conceição da Silva, José de Brito Lourenço-Júnior

**Affiliations:** 1 Postgraduate Program in Animal Science (PPGCAN), Institute of Veterinary Medicine, Federal University of Pará (UFPA), Castanhal 68746-360, Brazil; adrinysantos2@gmail.com (A.d.S.M.L.); thomazguimaraes@yahoo.com.br (T.C.G.d.C.R.); andregms@gmail.com (A.G.M.e.S.); andrea.vianacruz@gmail.com (A.V.d.C.); anapauladferreira0019@gmail.com (A.P.D.F.); joselourencojr@yahoo.com.br (J.d.B.L.-J.); 2 Institute of Animal Health and Production, Federal Rural University of the Amazônia (UFRA), Belém 66077-830, Brazil; jamileandrea@yahoo.com.br; 3 Centre for Interdisciplinary Research in Animal Health (CIISA), Faculty of Veterinary Medicine, University of Lisbon, 1300-477 Lisboa, Portugal; monicacosta@fmv.ulisboa.pt (M.M.C.); japrates@fmv.ulisboa.pt (J.A.M.P.); 4 Laboratório Associado para Ciência Animal e Veterinária (AL4AnimalS), Faculdade de Medicina Veterinária, Universidade de Lisboa, 1300-477 Lisboa, Portugal; 5 Nucleus of Agricultural Sciences and Rural Development, Federal University of Pará, Cametá 68400-000, Brazil; amqcunha@gmail.com; 6 Institute of Health Sciences, Federal University of Pará, Belém 66075-110, Brazil; vanessacosta@ufpa.br; 7 Statistics Department, Federal Rural University of the Amazônia (UFRA), Belém 66077-830, Brazil; profvinibarbo@gmail.com

**Keywords:** Amazon, liver composition, fat-soluble vitamins, lipid, rearing systems

## Abstract

The nutritional composition of bovine liver, particularly in terms of vitamins E, β-carotene, and cholesterol concentration, is significantly influenced by the cattle-rearing system and diet. This study aimed to elucidate the impact of four predominant cattle-rearing systems in the Eastern Amazon region on the vitamin E, β-carotene content, and cholesterol levels in bovine liver during the rainy season. Liver-tissue samples were collected from 48 cattle, with twelve specimens representing each rearing system. The systems encompassed two native pastures located in flood-prone areas, cultivated pastures on upland terrain and a confinement-rearing system. Our findings indicate that, when considering all rearing systems collectively, there were no significant differences in the levels of vitamins and cholesterol in the liver tissues (*p* > 0.05). However, a marked disparity in cholesterol levels emerged when comparing intensive rearing systems to extensive ones, with the former demonstrating notably higher concentrations (*p* = 0.01). Additionally, the intensive rearing system was associated with elevated levels of β-carotene (*p* < 0.01), α-tocopherol (*p* = 0.01), and β-tocopherol (*p* = 0.01) relative to the extensive systems. No significant variations were observed amongst the different extensive systems (*p* > 0.05). These results suggest that, while all rearing systems yielded liver tissues with typical concentrations of vitamins and cholesterol, the intensive rearing system led to a higher accumulation of certain vitamin compounds. This study highlights the substantial nutritional implications of different cattle-rearing systems in the Eastern Amazon and provides valuable insights for developing dietary strategies to optimize the nutritional quality of bovine liver. Therefore, the generated results are groundbreaking in the Eastern Amazon, Brazil, and inspire the development of new research projects to address other demands in this field and achieve additional outcomes.

## 1. Introduction

The endeavour for a balanced human diet often points towards the incorporation of animal-derived products, attributed to their high biological value and favourable impact on the immune system [1]. However, a significant portion of the population faces nutritional deficiencies, notably in vitamins, which can lead to issues with physical development, the onset of pathologies, and even premature deaths [2,3]. In this realm, beef liver emerges as a noteworthy product of animal origin, heralded for its nutritional potency, primarily as a source of readily available vitamins, and is also recognised for enhancing immunological responses [4,5,6]. Vitamins, along with other organic substances, are integral for human life regulation, serving as enzyme cofactors, regulatory elements, and active participants in metabolic processes [7].

Cholesterol, another pivotal component of the human diet, stands as one of the most recognised lipids, playing a crucial role as the precursor to steroid hormones (e.g., oestradiol and testosterone), in addition to being a structural component of cell membranes and a precursor to bile acids and vitamin D [8,9]. Nonetheless, its excessive consumption is associated with the onset of degenerative diseases, contributing to an increased predisposition towards cognitive deficiencies and damage to the blood–brain barrier (BBB) [10]. Moreover, there is a direct relationship between cholesterol (LDL cholesterol) and cardiovascular diseases, as this lipoprotein facilitates the transport of cholesterol through tissues; however, when in excess, it leads to the blockage of blood flow due to the action of atheroma plaques [11]. Understanding the correlation between animal diet and tissue composition paves the way for an essential characterization of husbandry systems. Livestock-rearing systems, such as cattle, can be categorized into intensive systems, known for higher productivity and the use of concentrated feed; semi-intensive systems with a combination of pasture and supplementation; and extensive systems, relying solely on pasture and being more susceptible to edaphoclimatic conditions [12]. The variance in the final product’s composition across these systems is significant, and its elucidation can aid in crafting a diet that aligns well with human nutritional requisites [13].

Building upon the foundation of recent research highlighting the significant influence of rearing systems on the composition of animal products, this study seeks to expand the understanding in this domain. Notable studies include [14], who documented variations in mineral content in soil, pastures, and the longissimus dorsi muscle of cattle; Ref. [15], who observed dietary impacts on meat composition; Ref. [16], who reported fluctuations in the mineral content of buffalo livers across different rearing systems; and [17], who identified variations in cholesterol levels in buffalo meat due to differing rearing practices. These studies collectively underscore the potential variability in animal product composition attributable to rearing systems.

In this context, our study aimed to investigate the specific impact of varying rearing systems in the Eastern Amazon on the nutritional profile of bovine liver, with a particular focus on the concentration of vitamins E, β-carotene, and cholesterol. We hypothesized that the rearing systems in this region significantly influence these key nutritional parameters in bovine liver, especially during the rainy season. The objective was to provide a comprehensive assessment of how these rearing systems affect the vitamin and cholesterol content in the bovine liver, thereby contributing to a deeper understanding of the relationship between livestock-rearing practices and the nutritional quality of animal-derived food products.

## 2. Material and Methods

This study was granted an exemption from formal review by the Ethics Committee on Animal Use (CEUA) at the Federal Rural University of the Amazon (UFRA), Belém, Pará, Brazil, under protocol number 1928240123, dated 8 January 2023, considering the utilisation of slaughtered animals.

### 2.1. Rearing Systems and Diets

For the investigations, data were collected from three pasture-farming systems (main extensive systems in Eastern Amazonia), and the intensive system (confinement) (Table 1): System 1 entails Marajó native pasture (flood areas) located in the mesoregion of Ilha do Marajó, Santa Cruz do Arari, in the northern region of the state of Pará, Brazil; System 2 encompasses Lower Amazon native pasture (flooded areas) situated in Monte Alegre, the western region of the state of Pará, Brazil; System 3 involves upland cultivated pastures (areas without flooding, augmented with high yielding forages) in São Miguel do Guamá, the northeastern region of the state of Pará, Brazil.

The finishing period for animals in extensive systems was 4 months as described by Silva et al. [17], and System 4 is an intensive breeding system (confinement), where the diet was administered for 135 days, consisting of cassava peel, *Panicum maximum* cv grass silage, Mombasa (roughage), and a concentrate based on soybean meal, barley, ground corn and a premix (core, soybean meal and urea) (Table 2 and Table 3).

### 2.2. Animals and Samples

For this study, analyses were performed on a farm per rearing system, totalling 48 male, castrated, Nelore crossbred cattle, with twelve per rearing system. The initial weight of the animals in the intensive system was 533 kg, and slaughter weights ranged between 410 and 628 kg. Tissue collection took place in commercial slaughterhouses, sourced from herds designated for meat production and marketing. Selection for slaughter was based on body condition, prioritizing animals with higher levels of fat, as determined by the owners.

The investigation employed liver samples from cattle across three pasture-rearing systems (extensive system) and one in confinement (intensive system) within the Eastern Amazon, finished during the region’s rainiest season occurring between January and June.

### 2.3. Diet Collection and Chemical Analysis

Diet samples from each evaluated pasture/system were meticulously collected at five distinct points (each encompassing 1 m^2^) and subsequently homogenized, following which a 1 kg sample was isolated for analysis at the Animal Nutrition Laboratory of the Federal University of Pará, Campus Castanhal. Additionally, diet samples from animals reared in confinement were gathered. It is important to note that the feed samples were collected at a single time.

The collected samples were subjected to drying in a forced ventilation oven at 60 °C for a duration spanning between 24 and 72 h, a procedure aimed at averting the loss of volatile compounds and alterations in chemical composition. Following the drying phase, samples were allowed to equilibrate to room temperature to mitigate humidity fluctuations before being ground to a fine consistency in a Willey mill, employing a 1 mm sieve, in preparation for chemical analysis.

The assessment of total nitrogen levels was executed utilizing the INCT-CA N001/1 methodology, with the micro Kjeldahl technique [18]. Finally, the crude protein is obtained by multiplying the nitrogen in percentage by the factor 6.25 (CP). Ash content was ascertained through the INCT-CA M-001/1 method, employing a muffle furnace set at 600 °C for a 4 h duration. Ethereal extract (EE) was determined according to INCT-CA G-004/1. Neutral detergent fibre (NDF) and acid detergent fibre (ADF) were assessed employing the respective methodologies INCT-CA F-001/1, INCT-CA F-002/1, INCT-AC F-003/1, and INCT-AC F-004/1, with subsequent corrections for protein and ash content as recommended by the National Institute of Science and Technology in Animal Science [19]. Non-fibrous carbohydrates were evaluated following the Sniffen method [20], and total digestible nutrients (TDN) were calculated employing the Clemson University equation: TDN = 93.59 − (ADF × 0.936).

### 2.4. Analysis of Vitamin E, β-Carotene, and Cholesterol

The analyses for vitamin E, β-carotene, and cholesterol were conducted by the methodology delineated by [21]. Initiating the procedure, 0.200 g of lyophilized liver were placed in two glass tubes with Teflon caps to minimize exposure to light and oxygen. Duplicate samples were placed in two Kimax tubes (16 mL), with 0.20 g of ascorbic acid and 5.5 mL of saponification solution (composed of 11% potassium hydroxide dissolved in 45% distilled water and 55% absolute ethanol) added to each. The samples were then briefly homogenized using a vortex mixer (for 10 s) to prevent fragment agglomeration. Following this, the air within the tubes was replaced with nitrogen to deter sample oxidation, and the tubes were agitated using a vortex mixer until the ascorbic acid was fully dissolved. Subsequently, the tubes were immersed in a water bath (80 °C for 15 min) with agitation set at 200 rpm. Post-heating, the tubes were cooled under running water (for 1 min) and supplemented with 1.5 mL of distilled water and 3 mL of *n*-hexane before undergoing further homogenization in the vortex mixer (for 2 min). The tubes were then centrifuged (at 2500× *g* for 5 min) to separate the *n*-hexane phases (organic and upper), which were transferred to new Kimax glass tubes. Within these new tubes, a small amount of anhydrous sodium sulphate was added (using a spatula) and homogenized using the vortex mixer (for 10 s). Upon completion of this stage, an aliquot of the *n*-hexane phases (upper) was filtered through a 0.45 µm hydrophobic syringe filter into 1.5 mL amber vials, which were then placed into the high-performance liquid chromatography (HPLC) apparatus for subsequent analysis.

The HPLC determination was carried out in the normal phase with direct saponification and extraction with n-hexane, as pre-stipulated elsewhere [21]. The HPLC was equipped with the Agilent Zorbax Rx-Sil (Santa Clara, CA, USA) column of a 5 µm particle diameter and 4.6 mm ID × 25 cm (20 °C). Its mobile phase was composed of hexane isopropanol (99:1), with a flow rate of 1.0 mL/min and an injection volume of 10 and 100 µL for α-tocopherol and α-tocotrienol (vitamin E) and 100 µL for cholesterol, beta-carotene, and other compounds.

The determinations of cholesterol, β-carotene, and vitamin E were carried out using the external standard method. The samples were injected separately, and their identification was based on a comparison of retention times. Therefore, four calibration curves were employed for each tocopherol and tocotrienol profile (α, β, γ, and ∆). As for cholesterol and β-carotene, a curve was determined for each, both, like the previous ones, based on the relationship between area and concentration. This methodology was applied both in the analyses of the livers and in the animals’ diet.

### 2.5. Statistical Analysis

The experimental design was developed in a linear model, where four livestock production systems were analysed, including three extensive systems (Monte Alegre, Santa Cruz do Arari, and São Miguel do Guamá) and one intensive system (confinement in Santa Izabel), during a season of the year (rainy period). The analysis of variance was performed using the R statistical software (R 4.3.1).

Statistical analyses of the variables, comparing livestock production systems in a completely randomized design (CRD), after checking the assumptions of normality and homoscedasticity, were conducted through parametric tests (parametric ANOVA with the Tukey–Kramer post hoc test) and non-parametric tests (Kruskal–Wallis ANOVA with the Dunn post hoc test), both with a significance level of 5%.

## 3. Results

The bromatological and chemical composition of the diets fed to crossbred Nelore cattle reared across diverse rearing systems in the Eastern Amazon is delineated in Table 4.

### Evaluation of Cattle-Rearing Systems in the Eastern Amazon

The Shapiro–Wilk test and Levene test indicated deviations from their assumptions (*p* < 0.05). Table 5 elucidates the distinctions in vitamin and cholesterol content across four distinct rearing systems within the Eastern Amazon. The direct comparison between extensive and intensive systems revealed a significant disparity. The intensive system exhibited higher levels of cholesterol (0.94 mg/g), β-carotene (3.59 µg/g), α-tocopherol (2.92 µg/g), and β-tocopherol (2.01 µg/g) compared to the extensive systems.

Specifically, the intensive system (IS) had a higher cholesterol content (0.94 mg/g) compared to the extensive systems (ranging from 0.36 to 1.13 mg/g). Similarly, a notable disparity was observed in β-carotene and α-tocopherol levels, with the intensive system showcasing higher levels, exhibiting *p*-values of <0.01 and 0.01, respectively. The β-tocopherol also varied significantly with a *p*-value of 0.01, displaying a higher level in the intensive system.

These findings suggest that the intensive rearing system was associated with higher levels of these nutritional components in the cattle’s liver compared to extensive systems.

## 4. Discussion

### 4.1. Comparison across Four Cattle Rearing Systems in the Eastern Amazon

In the comparative analysis across the four rearing systems, there was no variation observed in the composition of vitamins and cholesterol in the animals’ liver tissue, which can be linked to the similar concentrations observed in the three extensive systems (MA, SCA, and SMG), constituting a larger portion of the analysis. According to [16], total cholesterol, α-tocopherol, and γ-tocopherol from the livers of buffaloes (*Bubalus bubalis*) are primarily influenced by the relationship between extensive and intensive ecosystems. Nonetheless, the values observed align well with their respective system characteristics, demonstrating suitability for human consumption and validating their quality as a source of animal-based nourishment.

The current literature lacks studies examining the quantitative differences in fat-soluble vitamins across diverse animal-husbandry systems in the Amazon during the rainy season, thereby accentuating the significance of this work and advocating for further research in this domain. Such investigations will unveil additional insights and offer explanations concerning the variations in vitamin content in the livers of cattle from the Eastern Amazon, ultimately refining the formulation of human diets with enhanced accuracy.

Among the scant studies available, a notable one conducted by [17] evaluated vitamin and cholesterol content across four rearing systems and two characteristic periods (rainy and dry) in the Amazon with buffalos’ muscles (*longissimus lumborum*). Although significant variations were observed, the influence of seasonal factors, such as rainfall, pasture availability, and quality, muddled the clarity of the results.

Another conjecture suggests that despite nutritional variations, the diets across the rearing systems sufficiently met the animals’ nutritional needs, leading to common deposition patterns. On the other hand, studies indicate that production systems significantly impact the feeding behaviours of cattle, especially in animals primarily consuming pasture in their diet [22].

### 4.2. Assessment of Extensive Systems

The direct assessment between extensive systems revealed no significant differences, possibly due to similar soil and pasture conditions across varying locations within the Eastern Amazon. Even though the systems are tailored to the edaphoclimatic characteristics of the Amazon biome, the standardization of sample collection during the rainy season could have led to these uniform outcomes. Standardization in beef-cattle production, in addition to increasing efficiency, guarantees consistency in the nutritional quality of the final product and food safety [23].

Like the previous evaluation, there remains a void in studies examining the quantitative differences in fat-soluble vitamins between extensive animal husbandry systems in the Eastern Amazon during the rainy season. However, the congruent values across extensive systems, as observed in this study, signify adequacy for human health and affirm the quality similarity in products from cattle grazing in the Eastern Amazon.

### 4.3. Direct Comparison between Intensive and Extensive Systems

Beef liver, rich in nutritional value and renowned for its oxidizing function, serves as a crucial addition to the human diet, offering various health benefits [24]. This direct evaluation elucidated differences in vitamin and cholesterol content between the rearing systems.

The liver, being central to cholesterol regulation, contains substantial amounts of cholesterol 393 mg in 100 g of raw liver and 601 mg when grilled, as per the Brazilian Food Composition Table [25]. This substantial cholesterol content, compared to other beef parts, underscores the liver’s prominence as a cholesterol-rich organ.

Comparatively with the Brazilian Food Composition Table [25], the cholesterol levels (mg/g) in the bovine liver from extensive (0.85) and intensive (0.94) systems conform to the standards found in the liver and are acceptable for human consumption. Interestingly, these averages exceeded the cholesterol levels reported in buffalo muscle from extensive systems in the Eastern Amazon, according to [17], further corroborating the liver’s superiority in cholesterol content relative to muscle, even across different species.

Vitamin A, pivotal for visual processes, epithelial tissue integrity, and immune-system function, derives from carotenoids or provitamin A, which also possess numerous health benefits. The consumption of carotenoids has been associated with reduced risks of obesity, diabetes, and certain cancer types, alongside promoting intestinal mucosa health and positive intestinal microbiota changes [26,27,28].

Despite the high concentration of β-carotene in pastures across extensive systems, real-time consumption control poses a challenge due to the extensive grazing areas and unregulated management. The ABIEC data indicates that cattle reared in extensive systems accumulate twice as many carotenoids in their meat compared to those in confinement [29]. However, the intensive system in this study exhibited a higher β-carotene level of 3.59 µg/g, three times the value of the extensive system, as shown in Table 5. The β-carotene content in the extensively reared cattle was potentially influenced by supplementation in the intensive system (a strategy to not rely entirely on animal consumption over pastures with good or poor nutritional qualities/contents). Another possible explanation for the diminished levels of β-carotene in extensively reared cattle, despite the higher provitamin content in their pastures, may be attributed to the increased levels of ADF (acid detergent fibre—Table 4), NDF (neutral detergent fibre—Table 4), lignin, and cellulose, which cause gastrointestinal filling and interfere with the animal’s digestibility [30], as assessed in Table 4. It can be observed that there is a higher quantity of β-carotene in the analyses of extensive system pastures; however, the levels of ADF and NDF in the chemical composition are higher in this type of system when compared to the intensive system. In other words, the animal’s consumption of this pasture was hindered because the cattle consumed less due to gastrointestinal filling, causing a quicker “satiety” effect, along with a direct impact on digestibility.

The cost-effectiveness and high nutritional value to the human diet make beef liver a commendable dietary option, satisfying up to 30% of the daily vitamin A requirements at a lower cost [31].

Vitamin E, vital for antioxidant defence, has demonstrated immunity enhancement and viral pathogenicity reduction in epidemiological studies. Its supplementation in humans has shown increased resistance to respiratory infections, underscoring the importance of maintaining adequate vitamin and mineral intake for optimal immune-system function [32].

The intensive rearing system revealed the highest α-tocopherol value, doubling the amount found in the extensive system. Research generally indicates that ruminants fed with fresh forage-based diets tend to have higher concentrations of vitamin E, even without supplementation [32]. However, another study challenged this idea, asserting that there are no significant changes associated with the environmental and chemical properties of the pasture [33]. Furthermore, there is still limited evidence regarding ruminant meat linked to slaughter weight and concentrations of α-tocopherol [34]. Another study with lambs has already indicated a higher content in animals raised on concentrate-based diets compared to those on hay-based diets [35]. Another study confirms that α-tocopherol concentrations are conditioned by the concentrations provided in the animal’s diet [36].

The higher β-tocopherol amount (2.01 μg/g of liver) in the intensive system could be linked to its dietary availability, as reflected in Table 4. The β-tocopherol found in the muscles of buffaloes originating from the USA and Canada, consuming a diet based on concentrate and free-choice hay for 180 days, presented a quantity of 0.013 mg/g; that is, they appear to be lower than those found in the current research [37].

In different finishing systems for cattle, intrinsic factors such as age and body weight at slaughter can affect the metabolism and energetic characteristics of the muscle in these animals, after the post-mortem process [38]. This can be associated with the information obtained from the concentrations of vitamin E, β-carotene, and cholesterol with the data in Table 1, as these animals have a higher weight and lower age at slaughter. The storage capacity of this vitamin is proportional to the increase in age [39]. Another study on vitamin E identified a limiting factor, also considered in this research for other vitamin compounds, regarding access to green pastures, which are mostly available for short periods. This could be addressed with adjustments in feed correlated with seasonality, aiming to mitigate the lack of vitamin E in the diet [40]. Following the recommended dietary allowances (RDA), the daily intake for vitamin E is 15 mg/day, and for vitamin A (β-carotene), it is 4800 μg/day for adults, regardless of sex [41,42]. Therefore, the consumption of 100 g of raw beef liver from the intensive system, considering the ideal concentrations of β-carotene, vitamin E, and cholesterol, stands out as the most suitable option for human consumption, thus substantiating its nutritional importance. In summary, this discussion unveils the potential nutritional implications of different rearing systems in the Eastern Amazon, emphasizing the need for further research to elucidate the observed variations in the vitamin and cholesterol content of cattle liver. The results contribute valuable insights to optimize dietary formulations and promote an enhanced understanding of the nutritional quality of beef liver from different rearing systems in the region. The averages of cholesterol concentrations in bovine liver were lower than those reported in buffalo liver, where the highest cholesterol levels were found in extensive systems in the Eastern Amazon: Nova Timboteua (2.00 mg/g); Santarém (2.57 mg/g); and Marajó (2.31 mg/g) [17]. Even across different species, this previous comparison highlights the inferiority of bovine liver in terms of cholesterol when compared to buffalo.

These significant findings are considered pioneering in the Eastern Amazon region, Brazil, giving rise to the conception of new research projects aimed at addressing additional demands in the field of animal production and nutrition, as well as generating further results.

## 5. Conclusions

Our results reveal a clear distinction between extensive and intensive rearing systems, with the latter demonstrating a notably higher concentration of vitamin compounds, highlighting its nutritional superiority. Therefore, its inclusion in the diet, recommended by a professional, is indispensable. This comparative analysis provides valuable insights into subtle nutritional variations influenced by different cattle-rearing practices, offering a solid foundation for making informed dietary decisions, as well as recognizing the value in unique ecological contexts such as the Eastern Amazon.

Our research not only expands the knowledge base on the nutritional aspects of different rearing systems but also emphasizes the critical role of professional dietary advice for maximizing the health benefits obtained from consuming bovine liver. These significant findings regarding bovine liver and its distinct content derived from these four different rearing systems are considered pioneering in the Eastern Amazon, Brazil, and pave the way for future research on the intricate nutritional dynamics of cattle farming under various environmental conditions.

## Figures and Tables

**Table 1:** Description of rearing systems.

Item	Extensive Systems, Pará, Brazil			Intensive System
	Flooded Pasture	Flooded Pasture	Cultivated Pasture	
Location (city)	Monte Alegre	Santa Cruz do Arari	São Miguel do Guamá	Santa Izabel
Kõppen–Geiger	Am	Am	Am	Af
Rainfall (mm)	2.000	2.500	2.250	2.599
Temperature (average/year)	27	26	26	26
Humidity (average/year)	85	86	85	85
Rainy season	December– May	January– June	January– June	January– June
Dry season	August– October	September– November	September– November	July– December
Final weight (kg) *, -	450	410	550	628
Age (months) *	32	36	24	24
Pasture	*Leersia, Hymenachne e Oryza*	*Axonopus, Trachypogon, Paspalum e Eragrostis*	*Megathyrsus maximu* ^1^	Cassava peel; silage of *Panicum maximum* cv. *Mombaça*

^1^ During the rainy season, there was supplementation with palm-kernel cake (dry matter = 90.47; crude protein = 11.12; neutral detergent fibre = 69.87; acid detergent fibre 48.23; Ash = 4.61; ether extract = 11.64). Supplementation (0.5% of the animal’s live weight) occurs because the availability and quality of pasture are low during this period. * values in averages. - The weight of the animals was determined at the time of slaughter and provided to the authors.

**Table 2:** Ingredients and proportions of the premix diet for cattle raised in confinement.

Premix	Proportion (DM-%)
Core	31.26
Soybean meal	55.51
Urea	13.23
Total	100.00

DM = Dry matter; core: calcitic limestone, sodium chloride, ventilated sulphur, monocalcium phosphate, manganese oxide, probiotic additive, raspberry flavour, mixed herbal flavour, sodium bicarbonate, silicon dioxide, onion extract, grape seed extract, calcium iodate, sodium monensin, manganese oxide, zinc oxide, sodium selenite, cobalt sulphate, copper sulphate, iron sulphate, vehicle, vitamins (A, D3, E).

**Table 3:** Ingredients and proportions of the diet of cattle raised in confinement.

Ingredient	Proportion (%)	% DM	Amount (kg)
Cassava peel	15	38.33	39.13
Barley	8	27.67	28.91
Ground corn grain	60	88.00	68.18
Premix	8.41	99.00	8.49
Grass silage	8.59	31.66	27.13
Total	100		171.85

DM = Dry matter.

**Table 4:** Chemical composition of diets.

Item (%)	Extensive Systems			Intensive System
	Monte Alegre	Santa Cruz do Arari	São Miguel do Guamá	
DM	19.37	39.14	36.48	54.57
OM	90.34	91.92	84.76	89.92
CP	7.75	11.75	18.92	12.72
ADF	27.48	25.23	17.01	11.26
NDF	71.21	73.64	54.71	30.72
NFC	9.49	4.31	7.22	43.11
TDN	47.51	48.99	54.37	79.55
Ash	9.66	8.08	15.24	10.08
EE	1.9	2.23	3.91	3.37
β-Carotene *	2.13	3.12	1.2	0.19
α-Tocopherol *	4.13	2.8	18.45	2.68
α-Tocotrienol *	0.15	0.24	0.44	3.07
β-Tocopherol *	0.05	0.13	0.11	1.22
β-Tocotrienol + γ-Tocopherol *	0.19	0.03	0.19	0.66
γ-Tocotrienol *	**	**	0.9	**
δ-Tocopherol *	1.25	1.12	3.82	3.41

DM = dry matter; OM = organic matter; CP = crude protein; ADF = acid detergent fibre; NDF = neutral detergent fibre; NFC = non-fibre carbohydrates; TDN = total digestible nutrients; EE = ether extract. * The vitamin compounds are measured in µg/g. ** unsensitized compound in the samples (below detectable limits).

**Table 5:** Vitamins (A and E) and cholesterol in the livers of cattle in different rearing systems.

Item (µg/g)	Extensive Systems (ES)			IS ^1^	*p*-Value			SEM
	MA	SCA	SMG		IsxES ^3^	ES ^4^	Systems ^5^	
Cholesterol ^2^	1.13 ^b^	0.36 ^b^	1.05 ^b^	0.94 ^a^	0.01	0.13	0.55	0.41
β-Carotene	1.32 ^b^	0.89 ^b^	1.07 ^b^	3.59 ^a^	<0.01	0.45	0.36	1.22
α-Tocopherol	0.87 ^b^	0.96 ^b^	1.34 ^ab^	2.92 ^a^	0.01	0.31	0.77	1.57
α-Tocotrienol	1.38	0.83	0.71	1.06	0.60	0.38	0.67	0.40
β-Tocopherol	1.16 ^ab^	0.63 ^b^	0.67 ^ab^	2.01 ^a^	0.01	0.33	0.06	0.32

^1^ Intensive rearing system; ^2^ The cholesterol is expressed in (mg/g liver). SEM: standard error of the mean; ^3^ Comparative analysis between intensive system and extensive systems; ^4^ Direct evaluation between extensive systems (Monte Alegre; Santa Cruz do Arari; and São Miguel do Guamá); ^5^ Referring to the analysis of the four rearing systems. ^a,b^ in the line indicate significant differences.

## Data Availability

The original contributions presented in the study are included in the article, further inquiries can be directed to the corresponding author.
